# Enhancing Phenanthrene Degradation by *Burkholderia* sp. FM-2 with Rhamnolipid: Mechanistic Insights from Cell Surface Properties and Transcriptomic Analysis

**DOI:** 10.3390/microorganisms13112608

**Published:** 2025-11-16

**Authors:** Ying Zhai, Jiajun Ma, Guohui Gao, Yumeng Cui, Ming Ying, Yihe Zhao, Agostinho Antunes, Lei Huang, Meitong Li

**Affiliations:** 1 Tianjin Key Laboratory of Organic Solar Cells and Photochemical Conversion, College of Chemistry and Chemical Engineering, Tianjin University of Technology, Tianjin 300384, China; xiaozhai@stud.tjut.edu.cn (Y.Z.); martintin@stud.tjut.edu.cn (J.M.); gaoguohui@stud.tjut.edu.cn (G.G.); cuiyumeng@stud.tjut.edu.cn (Y.C.); ym@tjut.edu.cn (M.Y.); 2 CIIMAR/CIMAR, Interdisciplinary Centre of Marine and Environmental Research, University of Porto, Terminal de Cruzeiros do Porto de Leixões, Av. General Norton de Matos, s/n, 4450-208 Porto, Portugal; yihe.zhao@ciimar.up.pt (Y.Z.); aantunes@ciimar.up.pt (A.A.); 3 Department of Biology, Faculty of Sciences, University of Porto, Rua do Campo Alegre, s/n, 4169-007 Porto, Portugal

**Keywords:** phenanthrene, rhamnolipid, biodegradation, cell surface characterisation, transcriptome analysis

## Abstract

Various surfactants have been applied for the remediation of polycyclic aromatic hydrocarbon (PAH)-contaminated environments, but their roles in bioremediation remain controversial. This study focused on rhamnolipid (a typical surfactant) and *Burkholderia* sp. FM-2 (a high-efficiency phenanthrene-degrading bacterium), investigating its effects on phenanthrene solubilization and biodegradation by analyzing cell surface characteristics and gene expression differences. Results showed that low concentrations of rhamnolipid (20–120 mg/L) promoted phenanthrene degradation, while high concentration (400 mg/L) exerted an inhibitory effect. At 20–56 mg/L, rhamnolipid altered the bacterial surface morphology and functional groups, facilitated lipopolysaccharide release, enhanced cell surface hydrophobicity, and increased zeta potential. When the rhamnolipid concentration was 20 mg/L, the phenanthrene degradation rates of cytoplasmic enzymes, periplasmic enzymes, and extracellular enzymes produced by the bacterium reached over 98% after 15 days of enzyme system culture, demonstrating its role in promoting enzyme production and activity. Transcriptomic analysis revealed that 56 mg/L (1 CMC) rhamnolipid enhanced degradation through multi-pathway regulation of gene expression: upregulating the gene encoding protocatechuate 3,4-dioxygenase to strengthen benzene ring cleavage; increasing the expression of genes related to ABC transporters and protein transport to promote phenanthrene transmembrane transport; and activating genes involved in metabolic processes such as pyruvate metabolism and the tricarboxylic acid (TCA) cycle to enhance central carbon metabolic flux. This regulatory mode optimizes energy supply and redox balance, and indirectly improves phenanthrene bioavailability by modulating membrane structure and function.

## 1. Introduction

The contamination of soil and groundwater by polycyclic aromatic hydrocarbons (PAHs) poses a serious threat to human health and ecological systems [1,2]. In order to achieve more efficient biodegradation, the application of biosurfactants has been explored as a means of facilitating the removal of PAHs from polluted environments [3]. However, the use of surfactants is a highly controversial topic, with reports of both promoting and inhibiting PAH biodegradation [3,4,5]. For instance, Vaidya et al. recently conducted a study on synthetic surfactants, including Tween-80, Triton X-100, cetyltrimethylammonium bromide, and sodium dodecyl sulphate. These surfactants were found to impede the biodegradation of chrysene [6].

Rhamnolipid is one of the most representative of the many biosurfactants because of its broad and universal nature in the field of biodegradation [7]. Rhamnolipids are glycolipids consisting of a hydrophilic head group (rhamnose portion) and a hydrophobic tail group (lipid portion) linked by an O-glycosidic bond [8]. Research has demonstrated that rhamnolipids can promote the solubilisation of PAHs and enhance the biodegradation of PAHs [9,10,11]. Furthermore, it has been demonstrated that the process under discussion serves to enhance the desorption of pollutants from soil, thereby promoting the bioremediation of organic pollutants by increasing their bioavailability [12]. Recent studies have examined the impact of rhamnolipids on the biodegradation of PAHs, with a particular focus on the formation of rhamnolipid micelles, changes in the solubility of organic pollutants, and alterations in the adsorption efficiency of heavy metals [9,13,14]. It has been demonstrated that rhamnolipids can influence the surface active properties of bacterial extracellular membranes, such as cell surface tension, Cell Surface Hydrophobicity (CSH), LPS content, zeta potential, ultrastructure and functional groups [12]. These properties are essential for the biodegradation of PAHs.

PHE is a highly hydrophobic polycyclic aromatic hydrocarbon with low solubility in water, which makes it difficult for microorganisms to utilise when it exists in solid form. Research indicates that rhamnolipid enhances both the contact between *Burkholderia* sp. FM-2 and phenanthrene (PHE) and bacterial migration toward PHE-containing microenvironments, thereby increasing PHE degradation by the strain [12]. The aim of this study was to investigate the effect of rhamnolipid on the degradation of PHE by the bacterium *Burkholderia* sp. FM-2, and the changes in the surface properties of the strain during the degradation process. *Burkholderia* sp. FM-2, a typical PHE-degrading bacterium isolated from contaminated soil of the Xinjiang oilfield, was chosen as the subject of this study [15]. This study selected rhamnolipid as the main research object to investigate the effects of rhamnolipid addition on the growth of strain *Burkholderia* sp. FM-2 and the degradation of phenanthrene (PHE) during the PHE degradation process by FM-2. The influence of potential mechanisms based on alterations in microbial surface properties (cell surface tension, CSH, cell surface zeta potential, LPS content, cell morphology and functional groups) is also considered. On a global scale, the implementation of field studies and the integration of bioremediation processes utilising rhamnolipids remains in its infancy. However, documented cases have demonstrated the considerable potential of rhamnolipids in the context of PAH-contaminated environments [16,17].

In this study, we incorporated transcriptomic analysis to identify gene expression patterns under different treatment conditions, thereby detecting surfactant-induced changes in gene expression. Rhamnolipids, as anionic biosurfactants, are widely used in surfactant-enhanced bioremediation experiments [18]. However, the molecular mechanisms by which rhamnolipids influence phenanthrene degradation remain poorly understood and require further investigation at the transcriptomic level. Herein, we employed RNA sequencing (RNA-seq) to conduct comparative transcriptome analysis, examining differential gene expression in strain FM-2 when grown on phenanthrene with or without rhamnolipids. Furthermore, we thoroughly analyzed the biological functions of these differentially expressed genes to elucidate the cellular processes underlying phenanthrene biodegradation in the presence of rhamnolipids. This study contributes to an improved understanding of the mechanisms governing surfactant-enhanced biodegradation of polycyclic aromatic hydrocarbons.

## 2. Materials and Methods

### 2.1. Materials, Instruments and Bacterial Culture Conditions

#### 2.1.1. Chemicals and Experimental Apparatus

PHE (≥97%) was purchased from Energy Chemical Technology (Shanghai) Co., Ltd., Shanghai, China. Rhamnolipids (≥95%) were obtained from Ltd. Ruijie Biotechnology Co., Ltd. (Xi’an, China). This product is a natural mixture obtained through microbial fermentation, primarily composed of mono- and di-rhamnolipid congeners, and exhibits excellent surface activity and biodegradability. All other reagents (hexane, phenol and 2-thiobarbituric acid) were of analytical grade.

#### 2.1.2. Bacteria and Growth Conditions

*Burkholderia* sp. FM-2 is a strain of phenanthrene-efficient degrading bacteria obtained from oil-contaminated soil of Xinjiang oilfield in China by pre-screening in our laboratory [12]. The 16S rRNA gene was analyzed as described previously, with its nucleotide sequence from FM-2 deposited in GenBank (accession number: KM263605) [15].

Minimum medium (MM) formulation: each litre of distilled water contained 12.5 g Na_2_HPO_4_·12H_2_O, 2.04 g KH_2_PO_4_, 0.4 g (NH_4_)_2_SO_4_ and 0.1 g MgSO_4_. Inorganic salt medium: 1.5 g Na_2_HPO_4_·12H_2_O, 0.7 g MgSO_4_, 3.96 g (NH_4_)_2_SO_4_, 0.01 g yeast powder and 3.48 g KH_2_PO_4_ [19].

PBS buffer (g/L): 0.2 g KCl, 0.24 g KH_2_PO_4_, 8 g NaCl, 1.44 g Na_2_HPO_4_, 1 L of deionised water, pH 7.4. The pH of the medium was adjusted to 7 ± 0.2 with 1 N hydrochloric acid and sodium hydroxide. The above medium was autoclaved to be used for the subsequent experiments.

### 2.2. Surface Tension of Rhamnolipid in MM

The surface tension of MM with different concentrations of rhamnolipids was measured at 25 °C using a tensiometer (model Q200, Fangrui Instrument Co., Ltd., Shanghai, China) to determine the critical micelle concentration (CMC). The specific experiments were based on the method of Kourmentza et al. [19]. The experiment was repeated three times, and the mean value was calculated.

### 2.3. PHE Solubilisation Assay

The solubilisation of PHE was assessed in MM containing 0–400 mg/L rhamnolipid. For each treatment, PHE (9.0 mg) was dissolved in 5 mL of hexane and added to a 100 mL conical flask containing 30 mL of MM. Even with a rhamnolipid concentration of 400 mg/L, the PHE concentration would be well above the saturation value for its solubility. After evaporation of hexane, rhamnolipid concentrations (0, 20, 56, 120, and 400 mg/L) were added to the MM and the medium was placed in a shaking incubator (200 rpm) for 36 h. All samples were filtered through a 10 mL glass syringe containing glass wool to remove PHE particles and other solid impurities. To reduce emulsification during the subsequent solvent extraction, 3 mL of 1N HCl was added to each filtered aqueous sample to precipitate rhamnolipids. The entire phenanthrene degradation culture medium was extracted with 30 mL of hexane, followed by two additional extractions of the residual phenanthrene. The combined organic phases were dried over anhydrous sodium sulfate. The solvent was removed under reduced pressure using a rotary evaporator, and the residue was re-dissolved in hexane. The concentration of PHE in the hexane extract was measured at 254 nm by a visible spectrophotometer (UV756, Spectrum Instrument Co., Ltd., Shanghai, China). The concentration of dissolved PHE was determined by using a calibration curve of PHE in hexane.

### 2.4. PHE Degradation Experiments

The *Burkholderia* sp. FM-2 strain was cultivated in MM with PHE as the sole carbon source. Cultures were centrifuged at 8000× *g* for 5 min at 4 °C, and the cell precipitates were washed twice with phosphate-buffered solution (PBS). First, the cell pellet was resuspended in MM to prepare a bacterial suspension with a cell density of 10^8^ [20] colony-forming units (CFU) per milliliter, thus completing the preparation of cells for the experiment. Subsequently, the phenanthrene (PHE) degradation experiment was carried out: 30 mL of MM containing 300 mg/L PHE was taken, and rhamnolipids at concentrations of 0, 20, 56, 120, and 400 mg/L were added. Then, 2% (*v*/*v*, volume ratio) of the cultured *Burkholderia* sp. FM-2 strain was inoculated into the medium. The aforementioned medium was placed under shaken incubation at 25 °C and 200 rpm for 36 h. Cells were detected and monitored by measuring the absorbance (Abs) at a wavelength of 600 nm, and the bacterial growth rate was calculated based on the changes in absorbance at this wavelength. PHE was then extracted following the method referred to in our previous research [20]. The PHE degradation rate was determined using a gas chromatograph equipped with a flame ionization detector (FID). A 10-μL aliquot of the organic phase was analyzed using an HP-5 capillary column (30 m × 0.32 mm ID, 0.25 μm film thickness). The oven temperature was programmed as follows: hold at 80 °C for 1 min, then increase to 290 °C at a rate of 10 °C/min, and finally hold at 290 °C for 10 min. An uninoculated medium served as a sterile control. Control experiments with different PHE concentrations were conducted to assess the actual PHE recovery after extraction. The above method was modified from that reported by Tao et al. [21]. Similar methods for determining PHE degradation rates have also been reported in references [22,23,24]. The PHE degradation rate was calculated as follows: PHE degradation rate (%) = (C_1_ − D_2_)/C_1_ × 100%, where C_1_ is the PHE recovery in the control experiment after time td (%), and D_2_ is the PHE recovery in the degradation experiment after time td (%).

### 2.5. Characterisation of Cell Surface Properties

#### 2.5.1. Cell Surface Hydrophobicity and LPS Content

Following a 36-h biodegradation experiment as described in the previous section, cells were collected by centrifugation (10,000× *g*, 5 min), washed three times with PBS and resuspended in PBS. The cell suspension was adjusted to an OD600 of 0.200 ± 0.010, which was denoted as T0 (the initial optical density of the aqueous phase). Thereafter, 1 mL of this bacterial suspension was placed in a 1.5 mL centrifuge tube, 0.2 mL of xylene was added, and the mixture was vortexed for 1 min. After the mixture was allowed to stand at room temperature for 15 min to facilitate phase separation, 0.8 mL of the clear lower aqueous phase was promptly transferred to a new centrifuge tube, and its OD600 was measured, denoted as T (the optical density of the aqueous phase after mixing with xylene). Each set of experiments was repeated three times and averaged. Cell surface hydrophobicity (CSH) is the percentage of cells transferred into the hydrocarbon phase from the aqueous phase OD_600_ before and after mixing. The calculation was conducted as follows: CSH (%) = (T0 − T)/T0 × 100% [25].

The extraction of LPS was performed using whole cell lysis methods, as LPS is a component unique to the outer membrane of bacteria with a Gram-negative cell wall [2]. Firstly, in order to remove all interfering substances, the cell precipitates were washed twice with PBS and the cells were separated by centrifugation (8000× *g*, 10 min) each time. The washed cells were then resuspended in PBS and the OD_600_ was adjusted to 1.0. The cell suspension was centrifuged at 8000× *g* for 10 min and the supernatant was discarded. For LPS extraction, 0.3 mL of distilled water and an equal volume of 90% phenol were added to the cell pellet. The mixture was heated at 70 °C for 10 min using a magnetic stirrer for agitation. Subsequently, the mixture was rapidly cooled to 10 °C and centrifuged at 8000× *g* for 10 min. The supernatant was carefully aspirated with a pipette, and the LPS extraction was repeated by mixing the phenol phase at the bottom with 0.2 mL of distilled water. The supernatants of the two phenol aqueous extracts were mixed in a centrifuge tube, 0.1 mL of 20% NaCl and 6 mL of 95% ethanol were added, and placed at −20 °C to precipitate the LPS overnight. Centrifugation was performed at 8000× *g* for 10 min to separate the LPS precipitate from the aqueous phase. To the solid phase, 0.1 mL of 0.5 M NaCl and 1 mL of 95% ethanol were added and the LPS precipitation process was repeated. Finally, distilled water was added to the precipitate to prepare a 0.1 mL sample. The LPS concentration in the sample was measured and then converted to the LPS content of the strain (per mL of cell suspension) according to the method reported by Al-Tahhan et al. [26].

#### 2.5.2. Zeta Potential Measurements

To evaluate the impact of rhamnolipid concentrations (0, 20, 56, 120, and 400 mg/L) on bacterial surface charge, zeta potential measurements were conducted under controlled ionic conditions. The bacterial cells were pre-cultured in a phenanthrene (PHE) degradation medium with a PHE concentration of 300 mg/L. In order to minimize potential ionic interference, samples were appropriately diluted to lower molar concentrations prior to analysis. Bacterial cells of *Burkholderia* sp. FM-2 were harvested by centrifugation, resuspended in phosphate-buffered saline (PBS), and adjusted to an optical density (OD600) of 0.500. Zeta potential was determined at 25 °C using a dynamic light scattering instrument (Zetasizer Nano ZS90, Malvern Instruments Ltd., Malvern, UK) equipped with an imaging module [27].

#### 2.5.3. SEM Preparation

PHE degradation experiments were performed to obtain *Burkholderia* sp. FM-2 cells as described in Section 2.4. Cells cultured for 36 h were centrifuged at 8000× *g* for 10 min at 4 °C. The cell precipitates were washed three times with deionised water and then fixed with 2.5% (*v*/*v*) glutaraldehyde for 30 min at 4 °C. Cells were centrifuged, the supernatant was discarded, and the precipitates were washed three times with deionised water and dehydrated for 10 min in ethanol (70%, 90% and 100% [*v*/*v*]) [28]. The bacterial precipitates were suspended in tert-butanol at room temperature for 15 min. Cells were freeze-dried and coated with gold prior to SEM analysis using a field emission scanning electron microscope (Merlin Compact, Carl Zeiss Ltd., Oberkochen, Germany).

#### 2.5.4. FT-IR Spectroscopic Determination

For cell surface functional group analysis, strain cells were enriched as described in Section 2.4. Cell pellets were washed three times with PBS and lyophilised with a freeze-dryer. Bacterial cells were grown in MM without rhamnolipid as a control. FT-IR spectra of dried cells mixed with KBr at a ratio of 1:100 were analysed using an aTensor II FTIR instrument (Bruker Optics, Ettlingen, Germany) [28].

### 2.6. Extraction and PHE Degradation by Periplasmic, Cytoplasmic and Extracellular Enzymes

While *Burkholderia* sp. FM-2 has been shown to be effective in degrading PHE in previous studies, the enzyme degradation experiments will explore the effect of rhamnolipids on the enzymes (Periplasmic, Cytoplasmic and Extracellular enzymes) produced [29]. Enzyme extraction from different parts of the cell using osmotic shock has been studied in the past [27].

To evaluate the effect of rhamnolipids on periplasmic, cytoplasmic, and extracellular enzymes, 20 mg/L rhamnolipids were added to MM during biodegradation of PHE (300 mg/L) by *Burkholderia* sp. FM-2. The rhamnolipid-added cultures were placed in an oscillating incubator at 25 °C (200 rpm). After 3 days of incubation, the culture medium was centrifuged (6000 rpm, 10 min, 4 °C) to harvest cells for enzyme extraction. The extraction methods of the above three enzymes were reported by Xu et al. [29]. The protein concentrations of the S1 (extracellular), S2 (periplasmic), and S3 (cytoplasmic) fractions were determined using the Bradford method [30]. Appropriate volumes (10% for S1, 2% for S2 and S3) of the enzyme solutions were inoculated into fresh reaction mixtures based on their respective protein concentrations to ensure that comparable amounts of total protein were added to the reaction system for a fair assessment of their catalytic potential. These reaction mixtures were incubated at 25 °C and 200 rpm for 1, 2, 5, 10, and 15 days, with all experiments conducted in triplicate alongside control groups maintained under identical conditions.

### 2.7. Transcriptome Response of Burkholderia sp. FM-2 to Rhamnolipid Treatment

#### 2.7.1. RNA Purification and Transcriptome Sequencing

The cultured *Burkholderia* sp. FM-2 bacterial broth was inoculated into phenanthrene degradation medium (containing 300 mg/L phenanthrene) at a 2% inoculum ratio. Different concentrations of rhamnolipid were added to the culture medium, with a total of 4 treatment groups set up: the group without rhamnolipid addition (0 CMC) served as the blank control; three concentrations were selected for the experimental groups, namely 0.5 CMC (below the critical micelle concentration), 1 CMC (equal to the critical micelle concentration), and 8 CMC (above the critical micelle concentration), forming a gradient comparison of “low concentration—critical concentration—high concentration” [31]. The cultured bacteria were centrifuged at 10,000 rpm for 10 min at 4 °C for 72 h. The organisms were washed three times with pre-cooled PBS and the supernatant was discarded, the organisms were put into liquid nitrogen for freezing for 10 min, and the samples were stored in a refrigerator at −80 °C after freezing, for the extraction of RNA. The transcriptomic sequencing was performed by Majorbio (Shanghai, China) using the NovaSeq X Plus platform (Illumina Inc., San Diego, CA, USA). The company employs a standard prokaryotic transcriptome sequencing workflow. Firstly, the Ribo-Zero™ Plus Kit (Thermo Fisher Scientific, Waltham, MA, USA) is used to remove ribosomal RNA (rRNA) from the total RNA, thereby enriching mRNA. Subsequently, the enriched RNA is fragmented, reverse-transcribed (using random primers), and subjected to bridge PCR amplification to construct the Illumina sequencing library. Finally, the constructed libraries are sequenced on the Illumina NovaSeq X Plus platform using a PE150 strategy (Illumina Inc., San Diego, CA, USA).

Total RNA was extracted using the Tiangen RNAprep kit (Tiangen, Beijing, China) according to the instructions and genomic DNA was removed using DNase I (TaKara, Kusatsu, Japan). The integrity of the RNA was examined using agarose gel electrophoresis and the concentration and purity of the extracted RNA was examined using the Nanodrop2000 (Thermo Fisher Scientific, Waltham, MA, USA). RIN values were determined using a Bioanalyzer Agilent 2100 (Agilent Technologies, Santa Clara, CA, USA).

#### 2.7.2. Real-Time Quantitative Fluorescence PCR (qRT-PCR) Validation Analysis

qRT-PCR assays were run on a Q9600 series real-time fluorescence quantitative PCR instrument DEG (Bio-Gener Technology Co., Hangzhou, China). 2× RealStar Fast dye-based qPCR premix (GenStar, Beijing, China) was used as follows: 10 μL of 2× RealStar Fast SYBR qPCR-premix, 0.5 μL of forward primer (10 μM), 0.5 μL of reverse primer (10 μM), 0.4 μL of reverse primer (10 μM), and 0.5 μL of reverse primer (10 μM), 0.4 μL High/Low ROX Reference Dye and 1 μL DNA template. The optimal cycling conditions were pre-denaturation at 95 °C for 2 min, followed by 40 cycles of amplification, with each cycle including denaturation at 95 °C for 15 s, annealing at 54 °C for 20 s, and extension at 72 °C for 30 s; fluorescence signals were collected at the extension stage throughout the whole process [32]. The 2^−ΔΔCt^ method was used to calculate the relative expression levels of the target genes. All samples were run independently three times.

### 2.8. Statistical Analysis

All experiments mentioned above were set up with three biological replicates and data are expressed as mean ± standard deviation (SD). Data were analyzed, statistically processed, and plotted using Origin 2025, IBM SPSS Statistics 27, and GraphPad Prism 9.5. One-way analysis of variance (ANOVA) was utilized to assess the significance of differences between groups and *p* < 0.05 was set as the threshold for statistical significance.

## 3. Results and Discussion

### 3.1. Critical Micelle Concentration of Rhamnolipids

**Figure 1:**
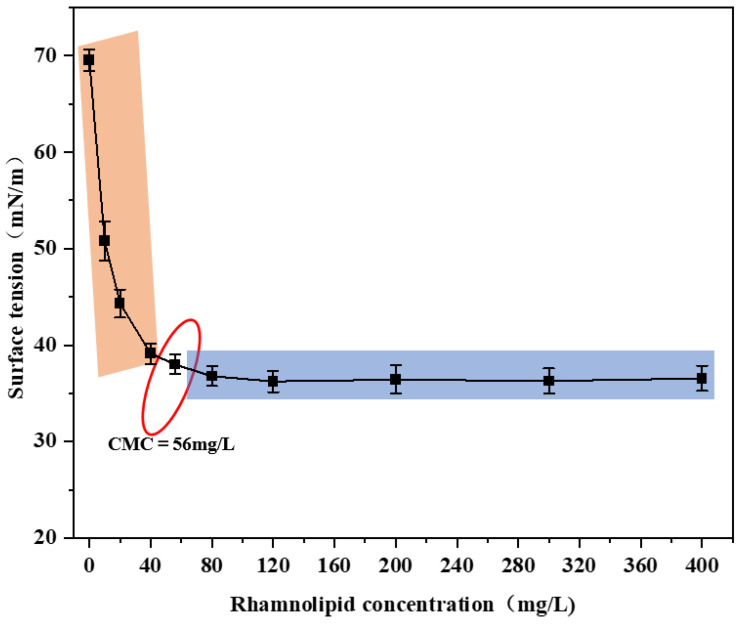
Surface tension variations during phenanthrene (PHE) degradation in the presence of rhamnolipid. The inflection point circled in red represents the critical micelle concentration (CMC) of rhamnolipid, which is 56 mg/L.

It is important to determine the CMC value of surfactants under different conditions, given their divergent propensity to solubilise PAHs [3]. The CMC values of surfactants in media and water may be different as the value is influenced by the electrolytes in the media [33]. The CMC of rhamnolipids was determined by plotting the relationship between surface tension and the concentration of rhamnolipid solution prepared in MM. As shown in Figure 1, the addition of rhamnolipids resulted in a rapid decrease in the value of the surface tension, and there was almost no decrease in the surface tension when the concentration of rhamnolipids was 56–400 mg/L. Therefore, the CMC of rhamnolipids in MM is determined to be 56 mg/L.

### 3.2. Effect of Rhamnolipid Concentration on Solubilisation of PHEs

Ben et al. reported on various biosurfactants assessed for their ability to enhance the apparent aqueous solubility of PAHs [34]. When the biosurfactant concentration exceeds the CMC, hydrophobic contaminants such as PHE can partition into the hydrophobic core of the micelles, thereby significantly increasing their apparent concentration in the aqueous solution [35,36,37].

**Figure 2:**
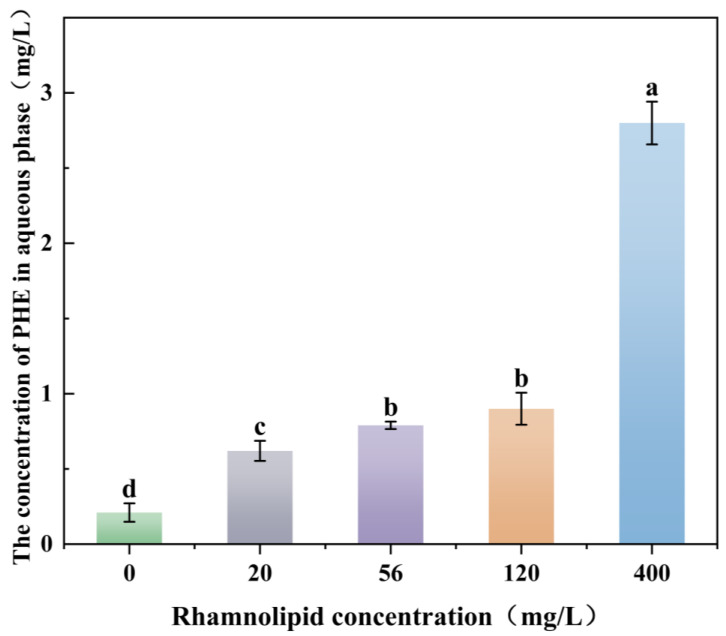
Solubilization Effect of Different Concentrations of Rhamnolipid on Phenanthrene. The different letters above the bars indicate significant differences among the different groups (*p* < 0.05, *n* = 3). Statistical significance was determined using Tukey–Kramer test.

The results of PHE solubilisation are shown in Figure 2. In the absence of rhamnolipids, the aqueous solubility of PHE was negligible (0.12–0.36 mg/L). At a rhamnolipid concentration of 56 mg/L (its CMC), the apparent solubility of PHE increased to 0.79 mg/L after 36 h, representing a 119.4% increase relative to the average control value, indicating a significant enhancement. When the rhamnolipid concentration was raised to 400 mg/L, the apparent solubility of PHE was further elevated to 2.8 mg/L after 36 h.

### 3.3. Effect of Rhamnolipids on PHE Biodegradation

Shin et al. [38] observed that the solubilization capacity of rhamnolipids for phenanthrene decreased with morphological transitions (from lamellae to vesicles to micelles), and that not all solubilized phenanthrene was available for microbial utilization. A previous study investigated the effect of nonionic surfactants on PHE degradation by strain GY2B (12 h biodegradation assay) and showed that low concentrations of TritonX-100 promoted PHE degradation, whereas high concentrations inhibited PHE degradation [39].

**Figure 3:**
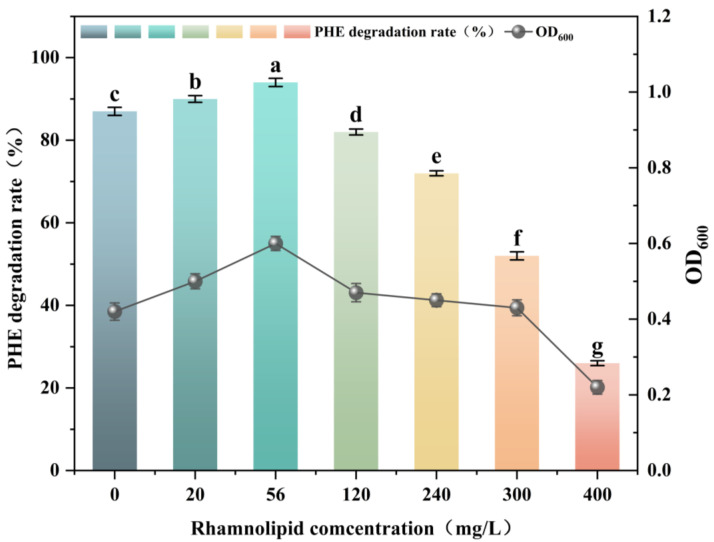
Effect of rhamnolipid on biodegradation of PHE by the bacterium FM-2 (36 h). The different letters above the bars indicate significant differences among the different groups (*p* < 0.05, *n* = 3). Statistical significance was determined using Tukey–Kramer test.

The effect of rhamnolipids on the removal efficiency of PHE by *Burkholderia* sp. FM-2 is shown in Figure 3. In the biodegradation system containing 20–56 mg/L rhamnolipids, the removal efficiency of PHE was more than 87% after 36 h. Notably, the highest PHE degradation rate was achieved at 56 mg/L rhamnolipid. Compared with the control experiment without added rhamnolipid, the PHE degradation rate increased by 11.96% after 36 h with the addition of 20 mg/L rhamnolipid. When the rhamnolipid concentration was 120–400 mg/L, the degradation rate of PHE decreased after 36 h, inhibiting the degradation of phenanthrene by strain FM-2. Makkar and Rockne [37] found that the addition of surfactants can actually inhibit the biodegradation of polycyclic aromatic hydrocarbons (PAHs) through toxic interactions, stimulation of surfactant-degrading bacteria, or sequestration of PAHs into surfactant micelles.

### 3.4. Effect of Rhamnolipids on Surface Hydrophobicity (CSH) and Release of Lipopolysaccharide (LPS)

**Figure 4:**
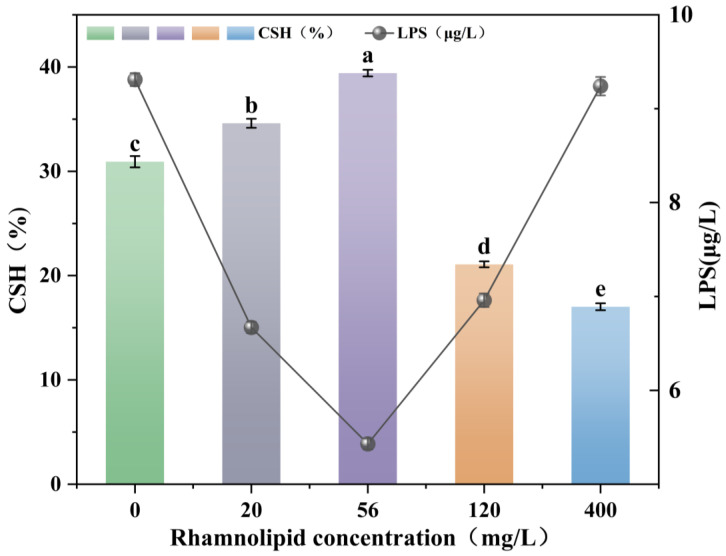
Effect of rhamnolipids on CSH and LPS (representative of cellular LPS content) of the bacterium FM-2 cultured for 36 h. The different letters above the bars indicate significant differences among the different groups (*p* < 0.05, *n* = 3). Statistical significance was determined using Tukey–Kramer test.

Surfactants have been demonstrated to either promote or inhibit the degradation of PAHs by strains, largely by altering cell surface hydrophobicity (CSH) and thus cell adhesion to hydrocarbons [40,41]. Chen and Zhu’s [42] findings indicated that the presence of surfactants resulted in the release of LPS (lipopolysaccharide) from the cells’ surfaces. This process rendered the cell surface of the strain more hydrophobic, thereby increasing the propensity for the accumulation of hydrophobic hydrocarbons on the cell surface. Therefore, an investigation was conducted into the effect of rhamnolipids on CSH and LPS content of *Burkholderia* sp. FM-2 (Figure 4), and its subsequent impact on PHE biodegradation.

In the absence of rhamnolipid, the CSH values at 36 h was 30.92%. The addition of low-to-intermediate concentrations of rhamnolipids (20 and 56 mg/L) significantly enhanced both CSH and PHE degradation. When the rhamnolipid concentrations were 20, 56 and 120 mg/L, the CSH values at 36 h of incubation were 34.6%, 39.41% and 21.08%. As the addition of 20 mg/L rhamnolipid enhanced the cell surface hydrophobicity (CSH) of the strain, the biodegradation efficiency of phenanthrene (PHE) in Section 3.3 was accordingly improved. This may be due to the interaction mechanism allowing the strain to utilise PHE in another way [5]. This observation aligns with the findings of Lin et al. [8] and Zhao et al. [43], who showed that high concentrations of rhamnolipids can significantly reduce CSH, thereby inhibiting the strain’s uptake and biodegradation of PHE. Some researchers have also found that low concentrations of rhamnolipids increase CSH in strains [44]. The contrasting effects of low versus high rhamnolipid concentrations underscore a concentration-dependent dual role: low concentrations promote PHE degradation by enhancing CSH and bacterial adhesion, whereas high concentrations inhibit degradation notwithstanding the enhanced solubilization, likely because PHE sequestration within micelles reduces its direct bioavailability to bacterial cells.

As illustrated in Figure 4, the effects of varying rhamnolipid concentrations on cell surface hydrophobicity (CSH) and lipopolysaccharide (LPS) release in *Burkholderia* sp. FM-2 were examined. The results demonstrated that as the rhamnolipid concentration increased, CSH initially rose and then declined, reaching a maximum value of approximately 39.41% at 56 mg/L. In contrast, LPS release showed an initial decrease followed by an upward trend with increasing rhamnolipid concentration. At the concentration of 56 mg/L, the released LPS measured 5.43 µg/L, accounting for 14.44% of the total cellular LPS [45]. This specific concentration (56 mg/L), which yielded the highest CSH and optimal PHE degradation, also represented a point of moderate LPS release, suggesting that a certain degree of LPS remodeling may contribute to the increased hydrophobicity.

At higher concentrations, such as 400 mg/L, CSH decreased, while LPS release remained relatively high. This inverse relationship at high concentrations suggests that excessive LPS release and other surface alterations disrupt cell envelope integrity and reduce CSH, ultimately compromising the strain’s ability to adhere to and degrade PHE effectively. These findings indicate that rhamnolipids influence surface properties by modulating LPS release, although changes in CSH are not exclusively governed by LPS and may involve additional surface structural factors [2].

### 3.5. Effect of Rhamnolipids on Surface Zeta Potential of Burkholderia sp. FM-2 Cells

**Figure 5:**
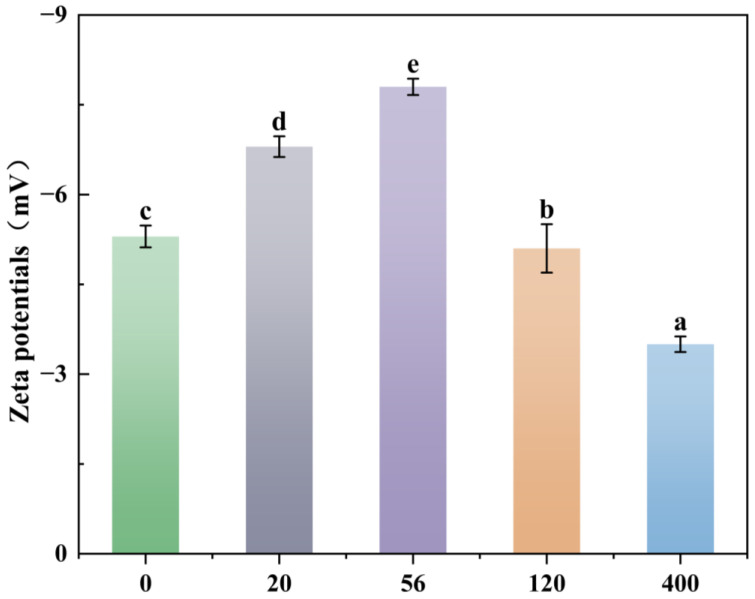
Variation of Zeta potential of cells cultured for 36 h treated with different concentrations of rhamnolipids. The different letters above the bars indicate significant differences among the different groups (*p* < 0.05, *n* = 3). Statistical significance was determined using Tukey–Kramer test.

Cells that had been cultured for 36 h were selected for zeta potential (ZP) analysis. As illustrated in Figure 5, rhamnolipids have been shown to exert a notable influence on the zeta potential within the degradation system. In the control group without rhamnolipid addition (0 mg/L), the zeta potential of the system stood at approximately −5.3 mV after 36 h of cultivation. Upon elevating the rhamnolipid concentration to 20 mg/L, the zeta potential ascended to around −6.8 mV. At 56 mg/L, the zeta potential hit a peak value of about −7.8 mV. Thereafter, as the rhamnolipid concentration continued increasing, the zeta potential exhibited a declining trend. When the concentration reached 120 mg/L, the zeta potential dropped to roughly −5.1 mV, and further increasing the concentration to 400 mg/L led to a subsequent decrease to approximately −3.5 mV [46]. This phenomenon indicates a tight linkage between zeta potential fluctuations and rhamnolipid (a biosurfactant) concentrations, meanwhile reflecting the intricate response patterns of negative charges on the cell surface to variations in rhamnolipid levels. Hua et al. [47] reported that biosurfactants increased the positive charge on *Candida antarctica* cell surfaces. In this study, the initial increase in surface negative charge (up to 56 mg/L) may be linked to alterations in the cell surface chemical structure or changes in the N/P ratio during phenanthrene degradation, as phosphate and nitrogen levels are known to modulate bacterial surface charge [48]. The subsequent decrease in negativity at higher concentrations (>56 mg/L) likely results from rhamnolipid molecules adsorbing to and effectively masking the cell surface.

### 3.6. SEM Analysis

**Figure 6:**
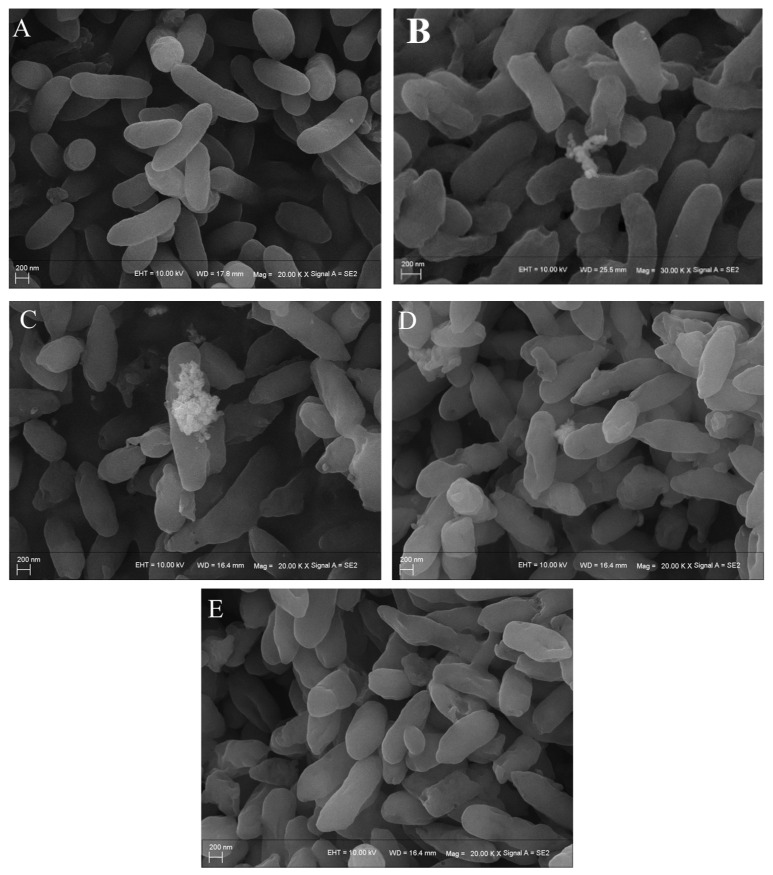
Scanning electron micrographs of *Burkholderia* sp. FM-2 cultured in MM for 36 h: (**A**–**E**) with 0, 20, 56, 120 and 400 mg/L rhamnolipid and phenanthrene (PHE), respectively.

While scanning electron microscopy (SEM) observations have documented the immobilization of *Burkholderia* sp. adherent biofilms by surfactants [49,50], the effects of rhamnolipids on the morphological changes (such as cell surface wrinkling and the production of extracellular polymeric substances) and substrate uptake patterns of the bacterium *Burkholderia* sp. FM-2 during growth on phenanthrene (PHE) remain poorly investigated. To examine the impact of rhamnolipids on the surface morphology of strain FM-2, this study observed bacterial samples treated with different concentrations of rhamnolipids using SEM. As shown in Figure 6A, cells without rhamnolipid addition exhibited a smooth surface, clear contours (short rod-shaped), and a plump, intact structure. With increasing rhamnolipid concentration, Figure 6B–E reveal the gradual appearance of wrinkles and roughness on the cell surface. We hypothesize that this morphological alteration, potentially induced by rhamnolipids, may enhance the interaction between bacterial cells and phenanthrene (PHE) by providing a larger surface area or modifying surface properties. Sotirova et al. [51] also observed altered surface morphology and folded cell membranes in *Bacillus cereus* cells treated with rhamnolipids, which is consistent with the findings of this study. Liu et al. [39] examined the morphology of *Sphingomonas sphaericus* under the combined effects of surfactants and PHE, finding that cells in the Brij30 group showed more severe shrinkage, roughness, and membrane damage compared to the TritonX-100 group. These results indicate that rhamnolipids exhibit low toxicity and mild effects on bacterial cells, which effectively explains the near-complete degradation of PHE within 36 h under the action of 56 mg/L rhamnolipids.

### 3.7. FT-IR Analysis

**Figure 7:**
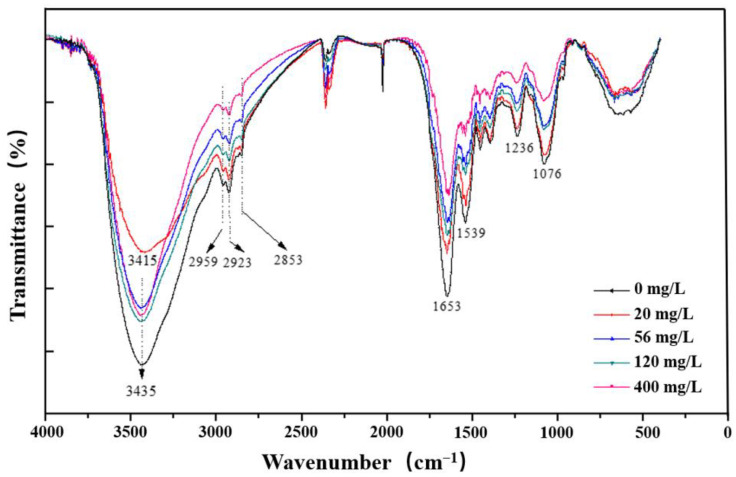
FTIR spectra of bacterium FM-2 in the presence of 0, 20, 56, 120 and 400 mg/L rhamnolipid for 36 h.

Fourier Transform Infrared (FTIR) spectroscopy has been widely used to confirm cell surface functional groups adsorbed by heavy metals [52], alkanes [53] and surfactants [46]. The functional groups on the surface of *Burkholderia* sp. FM-2 after degradation of PHE by the addition of different rhamnolipid concentrations were measured by FTIR spectroscopy as shown in Figure 7. The FTIR spectra showed that the surface of *Burkholderia* sp. FM-2 was altered by the addition of different concentrations of rhamnolipids. The broad absorption peaks correspond to the hydroxyl stretching vibrations of strained *Burkholderia* sp. FM-2 between 3500 and 3300 cm^−1^. At 56 mg/L rhamnolipid, the intensity of this peak was notably higher compared to the control group without rhamnolipid, suggesting that rhamnolipids might affect the exposure of hydroxyl groups on the cell surface, possibly through hydrogen bonding interactions. Similar results were reported for *Pseudomonas aeruginosa* biofilms after treatment with rhamnolipid [54] and Brij30-treated *Sphingomonas* sp. [39]. The peaks at 2923–2924 cm^−1^, attributed to the stretching vibration of the C-H group, also exhibited concentration-dependent changes. With the increase in rhamnolipid concentration from 20 mg/L to 56 mg/L, the intensity of these peaks gradually increased, implying that low concentrations of rhamnolipids could promote the synthesis or exposure of lipid-related substances on the cell surface, which is consistent with the reports by Zhang and Min [55].

Regarding the -COO^−^ groups, as stated by Yu et al. [56], the stretching vibrations of asymmetric and symmetric -COO^−^ groups usually occur in the ranges of 1660–1650 cm^−1^ and 1560–1539 cm^−1^, respectively. In our study, these vibrations were shifted to 1653 cm^−1^ and 1539 cm^−1^ when rhamnolipids were added, and the shift was most obvious at 56 mg/L rhamnolipid. This indicates that rhamnolipids may change the chemical environment of carboxyl groups through electrostatic interactions. Additionally, a peak at 1236 cm^−1^ (close to the previously mentioned 1234 cm^−1^) corresponding to the stretching vibration of -SO_3_^−^ was observed [20], and its intensity increased with the increase in rhamnolipid concentration, especially at 400 mg/L, suggesting a direct association between this group and the adsorption of rhamnolipids. Collectively, the addition of rhamnolipids led to alterations in hydroxyl, C-H, -COO^−^, and -SO_3_^−^ groups on the surface of *Burkholderia* sp. FM-2, and these changes were related to the concentration of rhamnolipids.

### 3.8. Effect of Rhamnolipids on Enzyme (Periplasmic, Cytoplasmic and Extracellular) Activities and Analysis of Enzyme Biodegradability

Many reports have shown that biosurfactants can stimulate the production of enzymes. Liu et al. [20] found that rhamnolipids increased the production of xylanase and CMCase. The addition of surfactants such as rhamnolipids can enhance microbial metabolism and enzyme accessibility to hydrophobic substrates, thereby facilitating the biodegradation of organic pollutants and the conversion of organic waste [57]. The degradation capabilities of the periplasmic, cytoplasmic, and extracellular enzyme pools were assessed by measuring the degradation rate of PHE in vitro, following the principle of measuring substrate depletion as described in standard protocols [58].

**Figure 8:**
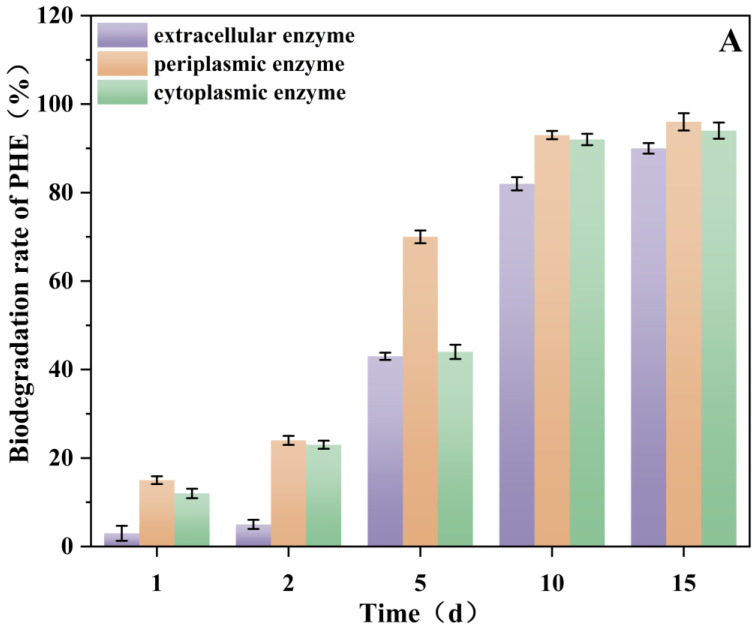

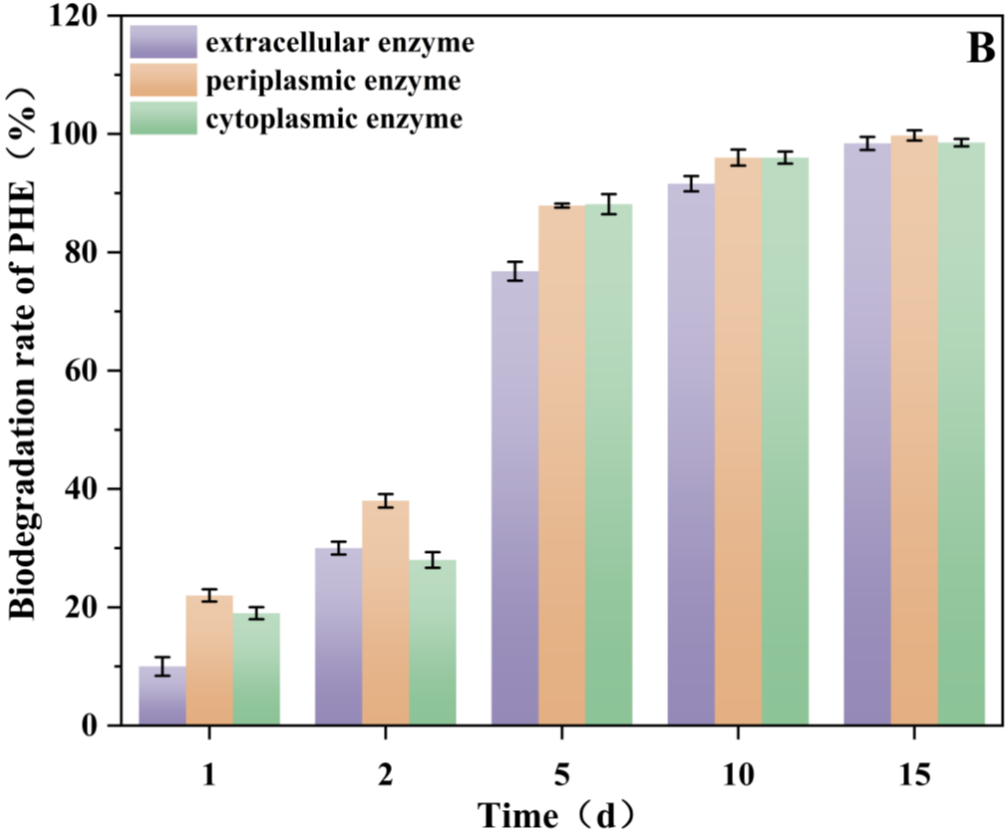
The degradation rate of PHE by cytoplasmic, periplasmic, and extracellular enzymes, (**A**) PHE degradation by enzyme extracts without rhamnolipids; (**B**) PHE degradation by enzyme extracts with 20 mg/L rhamnolipids.

Experiments on the degradation of PHE by enzymes were conducted in accordance with Section 2.6. Figure 8A,B show the catalytic degradation of PHE by periplasmic, cytoplasmic and extracellular enzymes of *Burkholderia* sp. FM-2 at different times. As shown in Figure 8A,B, the degradation rates of PHE after 5 d of incubation were increased by 17.9%, 44.12% and 33.8% in the presence of rhamnolipids (Figure 8B) compared to the control without rhamnolipids (Figure 8A), for periplasmic, cytoplasmic and extracellular enzymes, respectively. The results indicated that the addition of rhamnolipid increased the activities of the three enzymes of *Burkholderia* sp. FM-2. According to Zeng et al. [58], the increase in enzyme production caused by surfactants can be attributed to two main mechanisms. Firstly, surfactants increase the permeability of the cell membrane, which in turn increases enzyme excretion. Secondly, they stabilise enzyme activity. From 1 to 15 d, the degradation rates of PHE by the three enzymes followed an order of periplasmic ≥ cytoplasmic > extracellular enzyme. By comparing Figure 8B with Figure 8A, it can be observed that the phenanthrene (PHE) degradation activity of the cytoplasmic enzyme pool was significantly enhanced. This may be attributed to the specific upregulation of the expression levels or catalytic efficiency of key degradation enzymes, such as aromatic ring-hydroxylating dioxygenases. Following a 15-day incubation period, PHE was almost completely degraded by the three enzymes. The experimental approach of separately assessing the activity of enzyme pools from different cellular compartments, as employed by Xu et al. [29], demonstrates that their catalytic efficiency is highly substrate-dependent. Consistent with this, our results also revealed significant variations in phenanthrene degradation rates among the cytoplasmic, periplasmic, and extracellular enzyme pools of *Burkholderia* sp. FM-2. Deive et al. [59] reported that intracellular lipolytic activity was higher than extracellular enzyme pools activity, which is similar to the present study. In the present study, the periplasmic enzyme pools of *Burkholderia* sp. FM-2 degraded PHE more readily. The degradation rate of PHE by the enzyme pools showed an increasing trend to varying degrees with time. As shown in Figure 8B, the degradation rate of PHE by cytoplasmic enzyme pools was lower than that of other enzyme pools after 2 d of incubation, while the degradation rate of PHE by cytoplasmic enzyme pools was higher than that of the other two enzyme pools in 4–10 d, which may be due to the increase in enzyme activity or different metabolic mechanisms. The present study demonstrates that rhamnolipids promote enzyme production and enhance enzyme activity, thereby improving substrate degradation.

## 4. Transcriptome Response of *Burkholderia* sp. FM-2 to Rhamnolipid Treatment During Phenanthrene Biodegradation

### 4.1. Burkholderia sp. FM-2 Differentially Expressed Gene (DEG) Analysis

**Figure 9:**
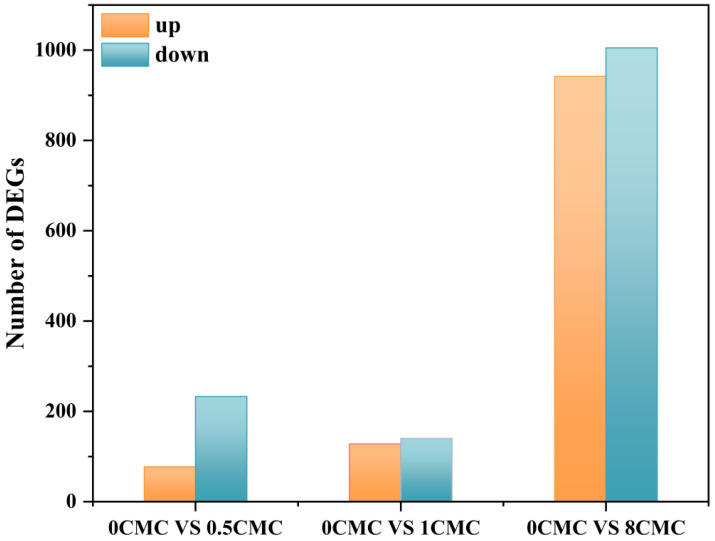
Summary of differentially expressed genes from comparison between the Rhamnolipid-treated samples and control samples.

Using transcriptome sequencing, we thoroughly investigated the gene expression profile of *Burkholderia* sp. FM-2 during phenanthrene degradation in the presence of varying concentrations of rhamnolipids. Transcriptomic comparisons were performed between a control group (0 CMC rhamnolipid) and each of the three rhamnolipid-supplemented groups. As shown in Figure 9, transcriptome analysis revealed pronounced dose-dependent alterations in gene expression. Relative to the control (0 CMC), the addition of 0.5, 1, and 8 CMC rhamnolipids resulted in 300 (77 up-/223 down-regulated), 268 (128 up-/140 down-regulated), and 1947 (942 up-/1005 down-regulated) differentially expressed genes (DEGs), respectively. The sharp increase in DEGs at 8 CMC—nearly seven times that of the 1 CMC group—implies a substantial stress response and possible metabolic disruption under high surfactant load, consistent with studies reporting surfactant-induced cellular stress at concentrations significantly above CMC [45]. Notably, although all tested concentrations were subtoxic based on growth assays, the 8 CMC treatment elicited widespread transcriptional changes indicative of cellular adaptation to stress, including the downregulation of key metabolic and biodegradation genes. In contrast, the 1 CMC treatment induced a more balanced and moderate transcriptomic shift with minimal antagonistic effects on central metabolic pathways. Given that prior degradation experiments confirmed optimal phenanthrene biodegradation at 1 CMC rhamnolipid, and considering the balanced gene expression profile (268 DEGs) without evident toxicity, we focused subsequent mechanistic analysis on the 1 CMC treatment to elucidate the role of rhamnolipids in enhancing phenanthrene degradation.

### 4.2. Functional Analysis of Differentially Expressed Genes

#### 4.2.1. GO Functional Annotation Analysis of Differentially Expressed Genes

**Figure 10:**
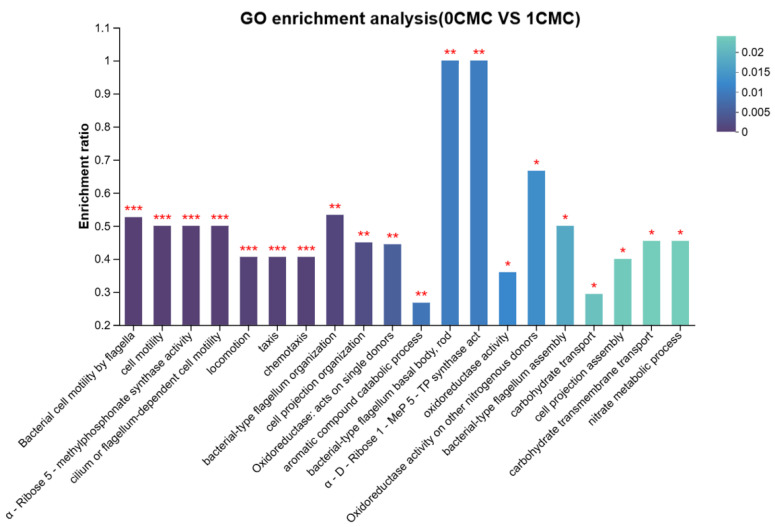
GO classification of DEGs in *Burkholderia* fungorum FM-2 in the three groups (0 CMC and 1 CMC). Colors indicate the significance of enrichment. By default, the redder the color, the more significantly enriched the GO term is. Specifically, FDR < 0.001 is marked with ***, FDR < 0.01 with **, and FDR < 0.05 with *.

To investigate the mechanism of rhamnolipid in the degradation of phenanthrene by *Burkholderia* sp. FM-2, this study analyzed the GO functional annotations (i.e., standardized classification annotations of gene functions from three dimensions: molecular function, biological process, and cellular component) of up-regulated and down-regulated genes based on the Gene Ontology (GO) database, and the relevant results are shown in Figure 10. When comparing the rhamnolipid-treated group (1CMC, 56 mg/L) with the control group, it was found that the differential genes mainly affected the transport and catalytic activities of transferases in FM-2 at the molecular function level, while also involving processes such as nucleic acid binding, as well as ribosomal and chromatin structural activities. From the perspective of biological processes, these differential genes were mainly annotated to metabolic activities, biosynthetic processes, and cellular processes; at the cellular component level, they were significantly enriched in processes such as organelles, ribosomes, cell membranes, and protein complexes.

#### 4.2.2. Gene Expression Associated with the Phenanthrene Degradation Pathway

Genes OI25_RS20545, OI25_RS20550, OI25_RS20490, and OI25_RS20495 are genes encoding aromatic ring hydroxylated dioxygenase, which plays an important role in the process of phenanthrene degradation. Aromatic ring hydroxylated dioxygenases are key enzymes in the aerobic bacterial metabolism of aromatic compounds, catalysing the stereospecific synthesis of chiral synthons and the biodegradation of aromatic pollutants [60]. The expression levels of the gene encoding this enzyme were up-regulated by 1.981-fold, 2.350-fold, 4.97-fold, and 6.824-fold after the addition of 1 CMC rhamnolipids (see Table S1). In addition to this, the gene encoding protocatechuate 3,4-dioxygenase (OI25_RS20535) demonstrated a 2.085-fold increase in expression following the addition of 1 CMC rhamnolipid, and this enzyme belongs to the family of aromatic ring-opening dioxygenases, which catalyse the ring-opening process of the benzene ring, and plays an important role in the process of phenanthrene degradation. The expression of the gene encoding aldehyde dehydrogenase (OI25_RS20520) was up-regulated 4.064-fold after the addition of 1 CMC rhamnolipids, which belongs to the family of aldehyde dehydrogenases and is responsible for the oxidation of aliphatic and aromatic, endogenous and exogenous aldehydes to form the corresponding carboxylic acids [61]. Data on the extent of gene expression in the phenanthrene degradation pathway are presented in Table S1.

#### 4.2.3. Transporter System-Related Differentially Expressed Genes

ABC transporter proteins are a class of ATP-driven pumps consisting of two transmembrane structural domains and two cytoplasmic side ATP-binding domains. They regulate the uptake of essential substances required for cell growth processes mainly through hydrolysis and binding of ATP [62]. Cobbett et al. [63] found that this protein regulates the uptake of organic substances by cells. In addition, ABC transporter proteins are also capable of inducing the excretion of intracellular substances against a concentration gradient, thus preventing the accumulation of metabolites in the cell that could have toxic effects on the cell [64]. After adding rhamnolipid at a concentration of 1CMC (56 mg/L), the differential gene expression levels of ABC transporters showed an increasing trend. This implies that rhamnolipids may stimulate the transmembrane transport of phenanthrene, thus promoting the increased biodegradation efficiency of phenanthrene by the strain (Table S2). In addition, the secondary metabolites produced after the addition of 1 CMC rhamnolipids could also be excreted through the ABC transporter protein system, a mechanism that could help to enhance the physiological activity of the bacterium, which in turn could positively affect the biodegradation efficacy of phenanthrene.

### 4.3. Validation of Differential Gene Expression via RT-qPCR

**Figure 11:**
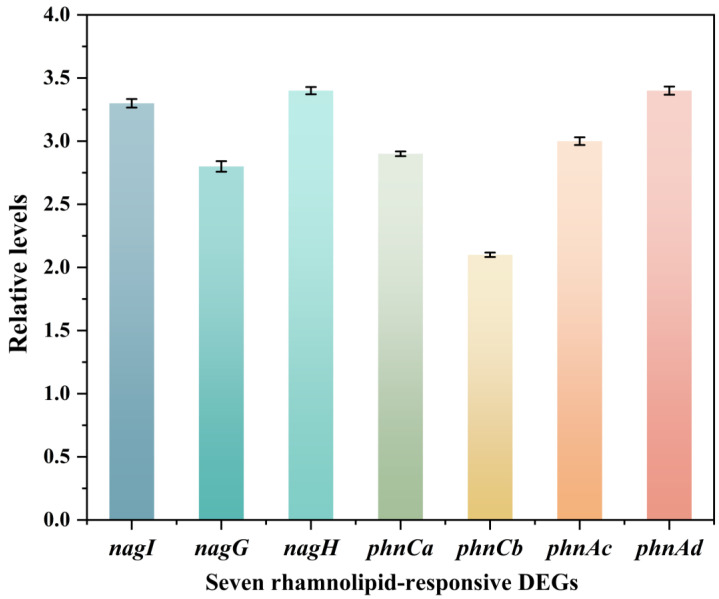
Relative expression levels of seven differentially expressed genes in response to rhamnolipids at 1CMC concentration after 36-h cultivation.

To validate the transcriptomic results, we used quantitative real-time PCR (qRT-PCR) to verify the expression levels of differentially expressed genes responsive to rhamnolipid. Seven genes encoding oxygenases were selected, namely *nagI* (encoding gentisate 1,2-dioxygenase), *nagG* and *nagH* (encoding salicylate 5-hydroxylase), *phnCa* and *phnCb* (encoding aromatic ring-opening dioxygenase), and *phnAc* and *phnAd* (encoding PAH dioxygenase). The analysis was conducted after 36 h of bacterial cultivation in the presence of 1 CMC rhamnolipid. As illustrated in Figure 11, the relative transcript abundances of these genes showed significant alterations, corroborating the RNA-seq data and highlighting their potential roles in the microbial response to rhamnolipid exposure.

### 4.4. Response Patterns of Major Metabolic Pathways

#### 4.4.1. Pyruvate Metabolism-Related Differential Gene Expression

Pyruvate plays an important pivotal role by linking the three major metabolic modules of glucose, fatty acids and amino acids through acetyl coenzyme A. Pyruvate in microorganisms is metabolised in a variety of ways [65]. However, most of them are catalytically converted to the core metabolite, acetyl coenzyme A with oxaloacetate, in the biooxidative pathway by specific enzymes, and subsequently involved in the TCA cycle. Differential genes associated with pyruvate metabolism were largely up-regulated by the addition of 1CMC rhamnolipids (Table S3), including those encoding NAD(P)-dependent alcohol dehydrogenase (OI25_RS25990), malate dehydrogenase (OI25_RS00420), acetyl-coenzyme A carboxylase carboxyltransferase (OI25_RS31645), acetaldehyde/hydroxy pyruvate reductase A (OI25_RS29005/29180), 2-isopropylmalate synthase (OI25_RS07535), phosphoenolpyruvate carboxykinase (GTP) (OI25_RS12255), aldehyde dehydrogenase (OI25_RS30445/28165/22605/12340/28995/06610), pyruvate dehydrogenase (OI25_RS19455), and acetyl coenzyme A/acetyltransferase (OI25_RS28190/33120/28220) genes. These upregulated genes reflect the adaptive strategy of *Burkholderia* sp. FM-2 for phenanthrene degradation with hamnolipid assistance, which relies on boosting central carbon metabolic flux. This regulatory pattern not only optimizes energy supply and redox balance but also likely enhances the bioavailability of phenanthrene indirectly by modulating membrane structure and function.

#### 4.4.2. Tricarboxylic Acid Cycle-Related Differentially Expressed Genes

The tricarboxylic acid cycle (TCA cycle) is a major pathway for energy acquisition in bacteria, and the FADH2 and NADH produced during the TCA process act as important donors in the electron transport chain to the oxidative phosphorylation process for oxidation into ATP that is available to the bacterium [66]. In addition, the TCA cycle supplies small molecules (e.g., succinate) for other biosynthetic pathways, which play an essential role in cellular life. Differential genes involved in the TCA cycle process are also largely up-regulated, mainly encoding the flavoprotein subunit of succinate dehydrogenase (OI25_RS31725), the iron-sulfur subunit of succinate dehydrogenase (OI25_RS31720), citrate synthetase (OI25_RS16505/39500), dihydrolipoic acid amide dehydrogenase (OI25_RS05370/19465), pyruvate dehydrogenase (OI25_RS05370/19465), and pyruvate dehydrogenase (OI25_RS05370/39500/19465), pyruvate dehydrogenase (OI25_RS19455), and phosphoenolpyruvate carboxykinase (OI25_RS12255) genes, and the gene difference multiplicity data are shown in Table S4. These results suggest that rhamnolipids may stimulate energy generation within strain FM-2, leading to enhanced metabolic activity and cellular proliferation, which could subsequently facilitate more efficient phenanthrene breakdown [31].

#### 4.4.3. Oxidative Phosphorylation-Related Differentially Expressed Genes

Oxidative phosphorylation refers to the release of energy during the gradual oxidation of organic matter, thereby promoting ATP synthesis. During oxidative phosphorylation, the electron transport chain can carry protons and electrons, transfer electrons from donors to acceptors, and carry protons between biological membranes, and the resulting electrochemical proton gradient can drive ATP synthesis [67]. Adding rhamnolipid at a concentration of 1 CMC (56 mg/L) significantly upregulates the expression of genes related to oxidative phosphorylation and ATP synthesis pathways, with a log2 fold change range of 1.050 to 6.706. This includes genes encoding the flavoprotein component of the succinate dehydrogenase complex, FAD-dependent oxidoreductases, and genes involved in NADH-quinone redox enzymes and cytochrome c (see Table S5 for a detailed list). These enzymes play a role in energy metabolic pathways and directly or indirectly influence intracellular oxidative phosphorylation processes and ATP production.

#### 4.4.4. Metabolic Transcriptional Response in Strain FM-2

**Figure 12:**
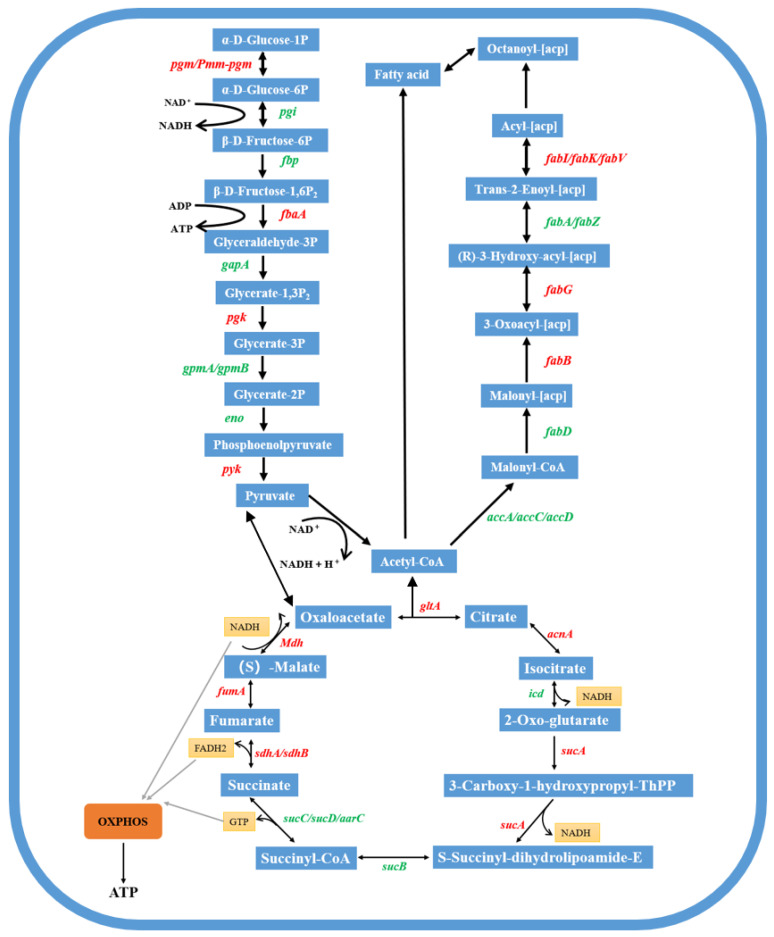
Schematic overview of gene expression of *Burkholderia* sp. FM-2 in response to rhamnolipid during phenanthrene (300 mg/L) biodegradation. Genes that were increased or repressed are shown in red and green font, respectively.

Genes involved in metabolic processes are the most prominently induced genomic genes by rhamnolipid treatment. A schematic overview of these genes, which are associated with metabolic pathways in *Burkholderia* sp. FM-2 during phenanthrene (300 mg/L) biodegradation under rhamnolipid stimulation, is presented in Figure 12.

## 5. Conclusions

This study systematically elucidates the concentration-dependent mechanism by which rhamnolipids affect the degradation of phenanthrene (PHE) by *Burkholderia* sp. FM-2. The results demonstrate that low concentrations of rhamnolipids (20–120 mg/L) can promote PHE degradation, while high concentrations (400 mg/L) exhibit an inhibitory effect. Within the rhamnolipid concentration range of 20–56 mg/L, rhamnolipids alter the bacterial surface morphology and functional groups, facilitate the release of lipopolysaccharides, enhance cell surface hydrophobicity, and increase the zeta potential. When the rhamnolipid concentration is 20 mg/L, after 15 days of cultivation of the enzyme system, the degradation rates of PHE by intracellular enzymes, periplasmic enzymes, and extracellular enzymes produced by strain FM-2 all reach over 98%, indicating that rhamnolipids can promote enzyme production and enhance enzyme activity. The improved degradation efficiency at the critical micelle concentration of rhamnolipids stems from integrated physiological and molecular responses, including changes in cell surface properties to enhance PHE adsorption, increased activity of key catabolic enzymes, and upregulated expression of genes related to aromatic compound degradation, central carbon metabolism, and ABC transporters. By combining surface characterization, enzyme activity determination, and comparative transcriptomics, this study conducts the first comprehensive analysis of rhamnolipid-induced metabolic remodeling during PHE degradation. It identifies 1 critical micelle concentration as the optimal concentration for balancing degradation efficiency and cellular homeostasis, and this finding provides important references for practical applications.

## Data Availability

The original contributions presented in this study are included in the article/Supplementary Materials. Further inquiries can be directed to the corresponding authors.

## Supplementary Materials

The following supporting information can be downloaded at: https://www.mdpi.com/article/10.3390/microorganisms13112608/s1, Table S1: DEGs associated with PAHs degradation in *Burkholderia* fungorum FM-2; Table S2: DEGs associated with transport in *Burkholderia* fungorum FM-2; Table S3: DEGs associated with pyruvate metabolism in *Burkholderia* fungorum FM-2; Table S4: DEGs associated with TCA cycle in *Burkholderia* fungorum FM-2; Table S5: DEGs associated with OXPHOS in *Burkholderia* fungorum FM-2.
