# Supplementary material for: Enhancing Phenanthrene Degradation by Burkholderia sp. FM-2 with Rhamnolipid: Mechanistic Insights from Cell Surface Properties and Transcriptomic Analysis
Source: Microorganisms. 2025 Nov 16;13(11):2608. doi: 10.3390/microorganisms13112608 (PMC12655266; doi:10.3390/microorganisms13112608)
Supplement: Supplementary file 1 [file microorganisms-13-02608-s001.zip › microorganisms-3917218-supplementary.pdf]

[Supplementary information]

Enhancing phenanthrene degradation by Burkholderia sp. FM-2 with rhamnolipid: Mechanistic insights from cell surface properties and transcriptomic analysis

Ying Zhai, Jiajun Ma, Lei Huang*, Meitong Li*

Affiliations:

College of Chemistry and Chemical Engineering, Tianjin Key Laboratory of Organic Solar Cells and Photochemical Conversion, Tianjin University of Technology, China.

***Corresponding Author**

E-mail address: huanglei@tjut.edu.cn (L. Huang) https://orcid.org/0000-0002-3490-3393

E-mail address: tjutlmt@email.tjut.edu.cn (M.T. Li)

Supplementary Sections

List of Tables

Table S1 DEGs associated with PAHs degradation in *Burkholderia* fungorum FM-2.

Gene ID	Gene description	Fold change*(log2ratio) log2 （0 CMC VS 1 CMC）
OI25_RS20510	sigma-54-dependent Fis family transcriptional regulator	1.071
OI25_RS20520	aldehyde dehydrogenase	4.064
OI25_RS20525	dihydrodipicolinate synthase family protein	1.587
OI25_RS20530	hypothetical protein	1.829
OI25_RS20535	protocatechuate 3， C4-dioxygenase	2.085
OI25_RS20540	2-hydroxychromene-2-carboxylate isomerase	1.934
OI25_RS20545	aromatic ring-hydroxylating dioxygenase subunit alpha	1.981
OI25_RS20550	aromatic-ring-hydroxylating dioxygenase subunit beta	2.350
OI25_RS20555	3-（cis-5%2C6-dihydroxycyclohexa-1%2C3-dien-1-yl）propanoate dehydrogenase	2.770
OI25_RS20470	LysR family transcriptional regulator	1.506
OI25_RS20475	2Fe-2S iron-sulfur cluster-binding protein	2.581
OI25_RS20480	gentisate 1%2C2-dioxygenase	3.564
OI25_RS20485	fumarylacetoacetate hydrolase family protein	4.212

OI25_RS20490	aromatic ring-hydroxylating dioxygenase subunit alpha	4.997
OI25_RS20495	aromatic-ring-hydroxylating dioxygenase subunit beta	6.824
OI25_RS20500	non-heme iron oxygenase ferredoxin subunit	5.410
OI25_RS20505	maleylacetoacetate isomerase	4.751

Fold change based on the log2 ratio of gene abundance from comparison between the rhamnolipid(1CMC) treated-samples and control sample (0 CMC).

Table S2 DEGs associated with transport in *Burkholderia* fungorum FM-2.

Gene ID	Gene description	Fold change*(log2ratio)
		log2 （0 CMC VS 1 CMC）
OI25_RS25415	branched-chain amino acid ABC transporter permease	2.513
OI25_RS36205	iron-siderophore ABC transporter substrate-binding protein	3.254
OI25_RS32210	ABC transporter permease	3.710
OI25_RS38585	ABC transporter permease subunit	7.795
OI25_RS38580	ABC transporter permease subunit	4.524
OI25_RS40505	ABC transporter permease	2.465
OI25_RS06175	histidine ABC transporter permease HisQ	2.416
OI25_RS39160	rhamnose ABC transporter substrate-binding protein	4.186
OI25_RS11255	ABC transporter permease subunit	2.080
OI25_RS32195	ABC transporter ATP-binding protein	3.237
OI25_RS09145	sugar ABC transporter ATP-binding protein	1.911
OI25_RS32035	sugar ABC transporter substrate-binding protein	2.111
OI25_RS05620	ABC transporter permease subunit	2.192
OI25_RS18315	ABC transporter ATP-binding protein	2.246
OI25_RS25425	ABC transporter ATP-binding protein	1.733

OI25_RS25420	branched-chain amino acid ABC transporter permease	1.478
OI25_RS36210	cyclic peptide export ABC transporter	4.146
OI25_RS35705	sn-glycerol-3-phosphate ABC transporter ATP-binding protein UgpC	1.859
OI25_RS23910	aliphatic sulfonate ABC transporter substrate-binding protein	2.567
OI25_RS26655	ABC transporter ATP-binding protein	2.427
OI25_RS36070	ABC transporter ATP-binding protein	2.453
OI25_RS37170	ABC transporter permease subunit	2.623
OI25_RS39145	sugar ABC transporter ATP-binding protein	4.784
OI25_RS40480	ABC transporter permease	4.484
OI25_RS25430	ABC transporter ATP-binding protein	2.286
OI25_RS06215	ABC transporter substrate-binding protein	2.093
OI25_RS26165	urea ABC transporter permease subunit UrtB	2.810
OI25_RS26160	urea ABC transporter permease subunit UrtC	2.352
OI25_RS39150	ABC transporter permease	6.600
OI25_RS39155	ABC transporter permease	5.099
OI25_RS32205	glycine betaine ABC transporter substrate-binding protein	3.641
OI25_RS32200	ABC transporter permease	3.434
OI25_RS38595	polyamine ABC transporter substrate-binding protein	3.871

OI25_RS38590	polyamine ABC transporter ATP-binding protein	7.645
OI25_RS36195	Fe（3+）-hydroxamate ABC transporter permease FhuB	3.442
OI25_RS37160	ATP-binding cassette domain-containing protein	3.280
OI25_RS29505	putative 2-aminoethylphosphonate ABC transporter ATP-binding protein	2.118
OI25_RS14455	ABC transporter ATP-binding protein/permease	1.902
OI25_RS39255	ABC transporter ATP-binding protein	2.350
OI25_RS05615	ABC transporter ATP-binding protein	5.368
OI25_RS05610	ABC transporter substrate-binding protein	2.002

Fold change based on the log2 ratio of gene abundance from comparison between the rhamnolipid(1CMC) treated-samples and control sample (0 CMC).

Table S3 DEGs associated with pyruvate metabolism in *Burkholderia* fungorum FM-2.

Gene ID	Gene description	Fold change*(log2ratio)
		log2（0 CMC VS 1 CMC）
OI25_RS09860	2-hydroxyacid dehydrogenase	1.122
OI25_RS31645	acetyl-CoA carboxylase%2C carboxyltransferase subunit beta	1.269

OI25_RS15255	acetyl-CoA carboxylase biotin carboxyl carrier protein	1.264
OI25_RS15250	acetyl-CoA carboxylase biotin carboxylase subunit	1.468
OI25_RS29180	glyoxylate/hydroxypyruvate reductase A	1.462
OI25_RS35785	alpha-hydroxy acid oxidase	1.496
OI25_RS25990	NAD（P）-dependent alcohol dehydrogenase	6.726
OI25_RS11775	MBL fold metallo-hydrolase	1.273
OI25_RS07535	2-isopropylmalate synthase	1.706
OI25_RS29570	thiamine pyrophosphate-requiring protein	1.384
OI25_RS30445	aldehyde dehydrogenase family protein	2.116
OI25_RS37080	thiolase family protein	2.216
OI25_RS28165	acetaldehyde dehydrogenase（acetylating）	3.108
OI25_RS22605	aldehyde dehydrogenase family protein	4.277
OI25_RS11535	glyoxylate/hydroxypyruvate reductase A	1.263
OI25_RS12340	aldehyde dehydrogenase family protein	3.072
OI25_RS18910	2-isopropylmalate synthase	1.482
OI25_RS34390	acetate--CoA ligase family protein	1.929
OI25_RS07120	fumarate hydratase	1.395
OI25_RS40275	acetate/propionate family kinase	0.671
OI25_RS28190	acetyl-CoA C-acyltransferase	2.638

OI25_RS12255	phosphoenolpyruvate carboxykinase（GTP）	1.007
OI25_RS29005	glyoxylate/hydroxypyruvate reductase A	1.934
OI25_RS28995	aldehyde dehydrogenase family protein	2.716
OI25_RS21050	phosphoenolpyruvate synthase	0.520
OI25_RS31745	malate dehydrogenase	1.905
OI25_RS33120	acetyl-CoA acetyltransferase	2.062
OI25_RS22740	acetate--CoA ligase	1.971
OI25_RS28920	NADP-dependent malic enzyme	1.239
OI25_RS19460	dihydrolipoyllysine-residue acetyltransferase	1.356
OI25_RS19465	dihydrolipoyl dehydrogenase	1.726
OI25_RS06610	acetaldehyde dehydrogenase（acetylating）	8.123
OI25_RS22610	methanol/ethanol family PQQ-dependent dehydrogenase	2.111
OI25_RS29525	cytochrome c	3.447
OI25_RS16795	ubiquinone-dependent pyruvate dehydrogenase	1.418
OI25_RS28220	acetyl-CoA C-acetyltransferase	2.357
OI25_RS00420	malate dehydrogenase	2.029
OI25_RS19455	pyruvate dehydrogenase （acetyl-transferring）%2C homodimeric type	1.163

Fold change based on the log2 ratio of gene abundance from comparison between the rhamnolipid(1CMC) treated-samples and control sample (0 CMC).

Table S4 DEGs associated with TCA cycle in *Burkholderia* fungorum FM-2.

Gene ID	Gene description	Fold change*(log2ratio)
		log2 （0 CMC VS 1 CMC）
OI25_RS05370	dihydrolipoyl dehydrogenase	1.687
OI25_RS17655	class II fumarate hydratase	1.015
OI25_RS31720	succinate dehydrogenase iron-sulfur subunit	1.027
OI25_RS16505	citrate synthase	1.003
OI25_RS07120	fumarate hydratase	1.395
OI25_RS31725	succinate dehydrogenase flavoprotein subunit	1.111
OI25_RS39500	citrate synthase	2.145
OI25_RS12255	phosphoenolpyruvate carboxykinase （GTP）	1.007
OI25_RS19455	pyruvate dehydrogenase （acetyl-transferring）%2C homodimeric type	1.163
OI25_RS31770	aconitate hydratase AcnA	1.003
OI25_RS19460	dihydrolipoyllysine-residue acetyltransferase	1.341
OI25_RS19465	dihydrolipoyl dehydrogenase	1.726
OI25_RS00420	malate dehydrogenase	2.029

Fold change based on the log2 ratio of gene abundance from comparison between the rhamnolipid(1CMC) treated-samples and control sample (0 CMC).

Table S5 DEGs associated with OXPHOS in *Burkholderia* fungorum FM-2.

Gene ID	Gene description	Fold change*(log2ratio)
		log2（0 CMC VS 1 CMC）
OI25_RS23070	polyphosphate kinase 2	1.266
OI25_RS00380	cbb3-type cytochrome c oxidase subunit I	1.388
OI25_RS00385	cbb3-type cytochrome c oxidase subunit II	2.871
OI25_RS22665	cytochrome c	1.913
OI25_RS36685	polyphosphate kinase 2	1.054
OI25_RS39225	F0F1 ATP synthase subunit beta	1.646
OI25_RS29540	cytochrome c oxidase subunit I	1.050
OI25_RS29545	cytochrome c oxidase subunit II	2.270
OI25_RS00535	cbb3-type cytochrome c oxidase subunit I	1.274
OI25_RS00530	cytochrome ubiquinol oxidase subunit II	1.588
OI25_RS33160	ubiquinol oxidase subunit II	6.706
OI25_RS38970	FAD-dependent oxidoreductase	1.489
OI25_RS31725	succinate dehydrogenase flavoprotein subunit	1.111
OI25_RS33165	cytochrome o ubiquinol oxidase subunit I	4.879
OI25_RS00545	cytochrome C oxidase subunit IV family protein	1.173
OI25_RS33175	cytochrome o ubiquinol oxidase subunit IV	3.316
OI25_RS33170	cytochrome o ubiquinol oxidase subunit III	4.186

OI25_RS07420	NADH-quinone oxidoreductase subunit M	1.589
OI25_RS07415	NADH-quinone oxidoreductase subunit NuoN	1.589
OI25_RS39205	cytochrome d ubiquinol oxidase subunit II	1.962
OI25_RS39200	cytochrome ubiquinol oxidase subunit I	1.666
OI25_RS25570	NADH-quinone oxidoreductase subunit M	1.870
OI25_RS00540	cytochrome c oxidase subunit 3	1.858
OI25_RS29535	cytochrome c oxidase subunit 3	1.460
OI25_RS11025	heme o synthase	1.110
OI25_RS10960	cytochrome ubiquinol oxidase subunit I	1.691

Fold change based on the log2 ratio of gene abundance from comparison between the rhamnolipid(1CMC) treated-samples and control sample (0 CMC).
